# Newly learned categories induce pre-attentive categorical perception of faces

**DOI:** 10.1038/s41598-017-14104-6

**Published:** 2017-10-25

**Authors:** Mengxia Yu, You Li, Ce Mo, Lei Mo

**Affiliations:** 1 School of Psychology, Beijing Normal University, Beijing, 100875 China; 2 Center for Studies of Psychological Application, South China Normal University, Guangzhou, 510631 China; 3 Peking-Tsinghua Center for Life Sciences, Academy for Advanced Interdisciplinary Studies, Peking University, Beijing, 100871 China

## Abstract

Face perception is modulated by categorical information in faces, which is known as categorical perception (CP) of faces. However, it remains unknown whether CP of faces is humans’ inborn capability or the result of acquired categories. Here, we examined whether and when newly learned categories affect face perception. A short-term training method was employed in which participants learned new categories of face stimuli. Behaviorally, using an AB-X discrimination task, we found that the discrimination accuracy of face pairs from different learned categories was significantly higher than that of faces from the same category. Neurally, using a visual oddball task, we found that deviant stimuli whose category differed from standard stimuli evoked a larger N170. Importantly, the visual mismatch negativity (vMMN), starting from 140 ms after stimuli onset, was stronger with the between-category deviants than with the within-category deviants under the unattended condition. Altogether, our study provides empirical evidence indicating that CP of faces could be induced by newly learned categories, and this effect occurs automatically during an early stage of processing.

## Introduction

One of the most remarkable abilities of the human visual system is recognizing individual faces quickly and efficiently. Many event-related potential (ERP) and magnetoencephalography (MEG) studies have investigated the time course of face processing and reported an early face-selective component peaking at approximately 170 ms after the stimulus onset (N170 or M170)^1,2^. Researchers proposed that the N170 is associated with the early structural encoding of faces^3–6^. Meanwhile, face perception is modulated by categorical information in faces (e.g., identity, race, and expression), i.e., discrimination of two faces is easier when the faces straddle a category boundary than when they belong to the same category^7^, which is a phenomenon known as categorical perception (CP). Here, we investigate how quickly the CP effect occurs during face perception.

CP has been implicating in the processing of facial expressions^8–11^, race^12^, gender^13^ and the identity of faces^7,14,15^. For example, Beale and Keil (1995) first reported that CP occurred in processing a morphed continuum between two familiar faces^7^. The authors used an AB-X discrimination task in which A and B were sequentially presented morphs followed by a target X that was identical to either A or B. The participants selected which morph (A or B) was identical to X. The discrimination accuracy was higher when A and B straddled the category boundary than when they were both from the same identity category. Moreover, face CP can occur early during the time window of the N170. For example, following the second face onset, the amplitude of N170 was larger when the two morphed faces stemmed from two different identities than that when the faces belonged to the same identity^16,17^. Studies exploring CP in facial expression have found similar results of N170^18^. However, it is unknown whether the observed CP of faces is a result of learned categories or humans’ inborn capability. Therefore, the present study examined CP of newly learned face categories and the time course of its occurrence.

In addition to the N170, the visual mismatch negativity (vMMN) can be used to measure the brain’s early response to visual stimuli, including faces. The vMMN, which is considered a negative component over bilateral occipito-temporal sites with a latency beginning at approximately 140 ms after the stimulus onset, is a reliable indicator for the evaluation of the brain’s automatic and pre-attentive detection of change in visual stimuli^19–22^. The vMMN can be elicited by changes in visual features, such as orientation^23^, color^24–26^, shape^27^, orientation changes in faces^28,29^, and facial expression^30,31^. For example, the vMMN that was elicited by blue deviant stimuli was significantly larger than that elicited by green deviant stimuli in Greek speakers who used two distinct words to represent light and dark blue, but no difference was found in English participants who used only one word^24^. Additionally, the vMMN was elicited by facial expressions after subtracting the ERP elicited by the standard stimuli (neutral faces) from that elicited by the deviant stimuli (sad faces or happy faces)^31^. Thus, the vMMN provides an ideal tool for determining whether CP that is induced by learned face categories occurs automatically during the early pre-attentive processing stage.

In the current study, we first examined whether newly acquired categories could affect individuals’ perception of human faces using an AB-X discrimination task, and then, we investigated whether face CP occurs automatically during the pre-attentive processing stage using a visual oddball task and electroencephalogram (EEG) recording. In addition, we used morphed faces as stimuli and employed an intensive short-term training paradigm^32,33^ in which participants learned to map face stimuli to artificial categories. In the behavioral discrimination task, we expected that after training, faces in different categories would be easier to discriminate than those in the same category. In the ERP task, we hypothesized that if newly learned categories automatically affect face perception during the pre-attentive stage, the vMMN for the deviant faces whose acquired category differs from that of the standard faces would be stronger than that for the deviants from the same category as the standard stimuli.

## Materials and Methods

### Participants

Twenty-three healthy undergraduate students (9 males and 14 females, mean age = 20.26 years, SD = 1.17) from South China Normal University participated in the behavioral discrimination tests and training phase. Twenty of these participants participated in the post-training ERP test. All participants were right-handed and had normal or corrected-to-normal vision. None of the participants had any history of neurological impairment or psychoactive medication use. This study was approved by the Academic Committee of the School of Psychology, South China Normal University and was carried out in accordance with approved guidelines and regulations. All participants provided written informed consent and were paid for their participation. Another participant who did not complete the training sessions were excluded from the study.

### Stimuli

#### Distinctiveness and beauty ratings

Thirty-five face images from an in-house database^34^ were adopted as candidate stimuli and rated in terms of distinctiveness^15^ and beauty on a 7-point scale (1 = least distinctive, 7 = most distinctive; 1 = least beautiful, 7 = most beautiful) by an independent group of 20 undergraduates. All images were grayscale Chinese male faces with the external contours removed and were randomly presented at the center of the screen. Two faces that were rated as least distinctive (Face A: 3.35, Face B: 3.15) and almost equally beautiful (Face A: 3.65, Face B: 3.45) were chosen as the stimuli for the formal experiments.

#### Classification

**Figure 1:**
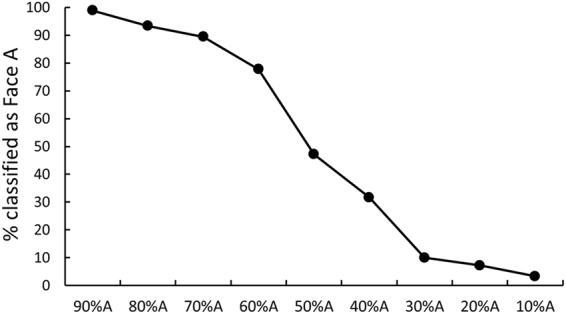
Classification of each morph from Face A to Face B. Nine morphed images with a 10% interval between the endpoint images (Face A and Face B) were presented in the classification task. Each point represents percentage that each morph was classified as Face A.

The two images were morphed (using FantaMorph version 5.4.1) to create a continuum. A classification task^15^ was carried out to assess the classification of the morphed faces and identify the category boundary of the continuum. An independent group of 30 undergraduates completed this task. Nine morphed images with a 10% interval between the endpoint images (Face A and Face B) were used as the stimuli in this task (e.g., 10% A/90% B, 20% A/80% B… 90% A/10% B). The two endpoints were presented side-by-side at the top of the screen, and a morphed image was presented at the center of the screen below the morphed image. The participants were asked to indicate which endpoint (left or right) most closely resembled the morph by pressing a key. The positions of the endpoint faces were counterbalanced. Each of the nine morphed images was presented five times, yielding a total of 45 trials. Figure 1 shows the mean classification of each morph from Face A to Face B. The categorical boundary was near the midpoint of the continua (50% A/B).

### Behavioral tests

Based on the classification of each morph and a pilot study, 12 pairs of morphs with a 25% interval between them were selected as the stimuli for the pre- and post-training behavioral discrimination tests. Half of the face pairs were more similar to Face A (“Face A pairs”, e.g., 5% B–30% B… 35% B–60% B), and half of the face pairs were more similar to Face B (“Face B pairs”, e.g., 40% B–65% B… 70% B–95% B). The trained faces (Face A, Face B, and 50% A/B) did not appear in the behavioral test. An AB-X alternative forced-choice paradigm^13,15^ was used to assess the participants’ discrimination of each pair of morphed faces. Following the presentation of a fixation cross for 750 ms, the target morph (X) was shown for 1,000 ms at the center of the screen, followed by a blank screen for 750 ms. The paired morphs (A or B) were then shown for 2,500 ms or until a response was made, followed by a 1,000 ms inter-trial interval. The target morph (X) was identical to either A or B. The participants were asked to indicate which test morph (A or B) was identical to the target by pressing a key as quickly and accurately as possible. After several practice trials, each pair was presented four times, and X was identical to A and B twice. The positions of A and B were counterbalanced. Twelve pairs were presented, yielding a total of 48 trials. The order of the presentation of the trials was random.

### Training phase

23 participants received intensive training in which they learned to map the three faces to two categories symbolized by two semantically irrelevant Chinese monosyllables. The participants were divided into two groups. During the training, Group 1 was instructed to assign Face A and 50% A to the **áng** category and Face B to the **duān** category, whereas Group 2 was instructed to assign Face A to the **áng** category and 50% A and Face B to the **duān** category. Hence, each endpoint face and the average face served as both a between-category pair and a within-category pair, which further minimized the interaction between the perceptual dissimilarity and category relationship. The training phase consisted of 7 separate 20- to 30-minute sessions that occurred twice per day. The first training session began immediately after the pre-training discrimination task. The post-training discrimination task was performed after the sixth session on the third day, and the ERP task was performed after the seventh session on the fourth day. The following four types of training tasks were employed: listening, naming, matching and judgment. In the listening task, the participants viewed one of the three faces and passively listened to the corresponding names, which were simultaneously presented. In the naming task, the participants were asked to orally pronounce the name of each face that was presented on the screen. In the matching task, the participants determined whether the sound they heard matched the face that was simultaneously presented. In the judgment task, the participants determined whether the face that was presented on the screen was **áng** or **duān** by pressing a key. The accuracy in the matching and judgment tasks was recorded for each participant to assess the training effect.

### ERP task

After the training, in addition to the behavioral discrimination task, the participants performed a visual oddball task while ERPs were recorded. This task consisted of 4 blocks of 540 trials. Each block included one deviant stimulus (Face A or Face B, two blocks each), and the average face (50% A) served as the standard stimulus in all trials. There were three types of trials in each block. In total, 80% of the trials were “standard” trials in which the standard stimulus was presented at the center of the screen with a fixation cross (“+”), while 10% of the trials were “deviant” trials in which a deviant was presented with the central fixation cross. The remaining 10% of trials were “target” trials in which the standard stimulus was presented with a fixation circle (“○”) instead of a cross. The participants were asked to fixate on the center of the screen and press the spacebar with their left or right index finger when they detected a fixation circle (“target” trials). During each trial, the face image was presented at the center on a gray background for 200 ms, followed by a blank screen jittered between 750 ms and 950 ms. The face image was 200 × 240 pixels and was presented on a 19-inch cathode-ray tube monitor with a resolution of 1024 × 768 pixels and a refresh rate of 75 Hz at a viewing distance of 80 cm. The stimulus order was pseudorandomized, and no consecutive deviant trials occurred in each block. The first five trials in each block were always standard trials. The block order was counterbalanced across participants. The required finger responses were also counterbalanced across participants.

### EEG recording and analysis

The EEG signals were recorded from 64 Ag/AgCl scalp electrodes that were placed equidistantly according to the international 10–20 system (QuickCap 64) using SynAmps 2 (Compumedics Neuroscan, Inc., Charlotte, NC). The vertical electrooculograms (VEOGs) were recorded using two electrodes that were placed above and below the left eye, while the horizontal electrooculograms (HEOGs) were recorded using two electrodes that were placed at the bilateral outer canthi of the eyes. All electrodes were referenced to the tip of the nose, and the impedance was maintained at 5 kΩ or below. The EEG recordings were sampled at a rate of 1,000 Hz and filtered offline with a 0.8–40 Hz bandpass zero-phase FIR digital filter (slope 24 db/Oct). The epochs ranged from −100 to 800 ms after the onset of the stimulus. Epochs containing a signal amplitude that exceeded ±100 μV were considered artifacts and excluded from the subsequent analyses. Finally, the data epochs were sorted and averaged to obtain the averaged waveforms that corresponded to the following four event types: standard, target, between-category deviant and within-category deviant. The percentage of the included trials after the artifact rejection was similar across the conditions (Mean ± SD: between-category, 78% ± 15%; within-category, 78% ± 16%; standard, 77% ± 14%; target 74% ± 17%), and no significant discrepancies were observed among the different conditions (One-way repeated-measures ANOVA: *F*(3,57) = 2.106, *p* = 0.147, η_*p*_^2^ = 0.100).

Based on previous studies^29,30^ and a scrutiny of the present N170, P2 and MMN distributions, the lateral posterior electrode sites (P5/6, P7/8, PO5/6, and PO7/8) were selected as the regions of interest (ROI) for the statistical analysis. The amplitudes and peak latencies of N170 and P2 were measured between 140 ms and 200 ms and between 200 ms and 300 ms, respectively. The mean amplitudes and latencies were analyzed using a repeated-measures ANOVA with the stimulus type (between-category deviant, within-category deviant, and standard) as the within-subject factor. In addition, the vMMN waveforms were obtained by subtracting the ERPs in response to the standard stimuli from those in response to the deviant stimuli. According to previous studies^28,31^, the mean amplitudes of the vMMN waveforms in the same ROIs as the N170 were measured separately across consecutive 70 ms latency windows within a 140–350 ms latency range (140–210 ms, 210–280 ms, and 280–350 ms). One-sample t-tests were performed to determine whether the mean amplitudes of the vMMN were significantly different from zero. Then, paired t-tests were conducted with stimulus type (between-category deviant vMMN and within-category deviant vMMN) as the within-subject factor. The degrees of freedom were adjusted using the Greenhouse-Geisser correction to account for nonsphericity.

### Data availability

All data generated or analyzed during this study are available from the corresponding author upon reasonable request.

## Results

### Training and behavioral data

**Figure 2:**
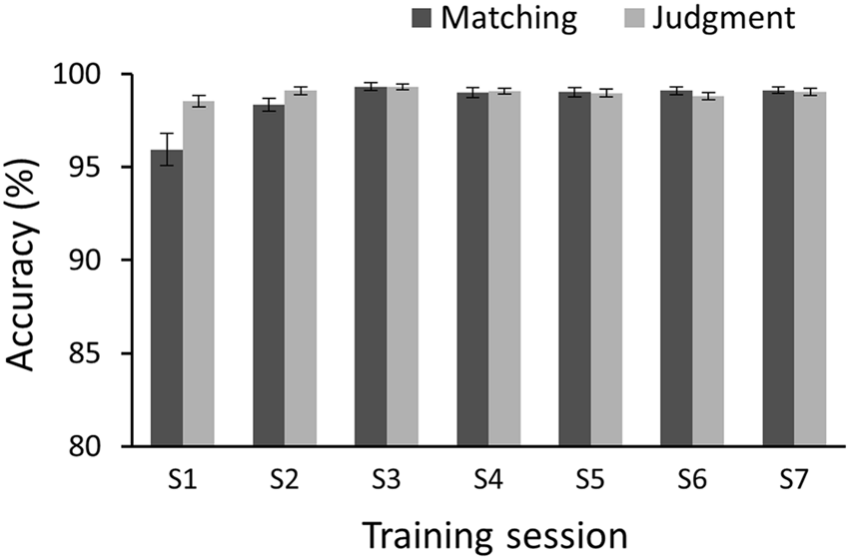
Participants’ mean accuracies in the matching and judgment tests during the training phase. Error bars represent the standard error of the mean.

The training data showed that all participants achieved highly satisfactory performance in the matching and judgment tests (see Fig. 2), with a mean accuracy above 98% in each session (except for matching session 1: 95.9%), indicating that the participants successfully learned to map the face stimuli to the artificial categories after the training phase.

For the behavioral discrimination tests, the discrimination of each pair during the pre- and post-training tests was averaged across the participants. For the pre-training test data, a two-way mixed ANOVA was conducted using face category (“Face A pairs” vs. “Face B pairs”) as the within-subjects variable and group (Group 1 vs. Group 2) as the between-subjects variable. The participants’ discrimination of “Face A pairs” and “Face B pairs” was similar (main effect: *F*(1, 21) = 0.199, *p* = 0.660, η_*p*_^2^ = 0.009), and there was no significant difference between the two training groups (interaction: *F*(1, 21) = 1.439, *p* = 0.244, η_*p*_^2^ = 0.064; main effect of group: *F*(1, 21) = 0.044, *p* = 0.836, η_*p*_^2^ = 0.002); thus, the data from the two groups were pooled together for further analyses. In addition, for the pre-training test data, we conducted a paired t-test to compare the accuracy of the discrimination of the face pairs that straddled the category boundary (i.e., 30–55%, 35–60%, 40–65%, and 45–70%) and the face pairs from the same identity category (i.e., other eight pairs). No significant differences were observed (*t*(22) = 1.824, *p* = 0.082, Cohen’s *d* = 0.38, 95% confidence interval [CI] for the difference: −0.11 to 0.01), which was consistent with previous findings that CP was not observed with unfamiliar morphed faces without preexposure to the endpoints of the continuum^15^.

**Figure 3:**
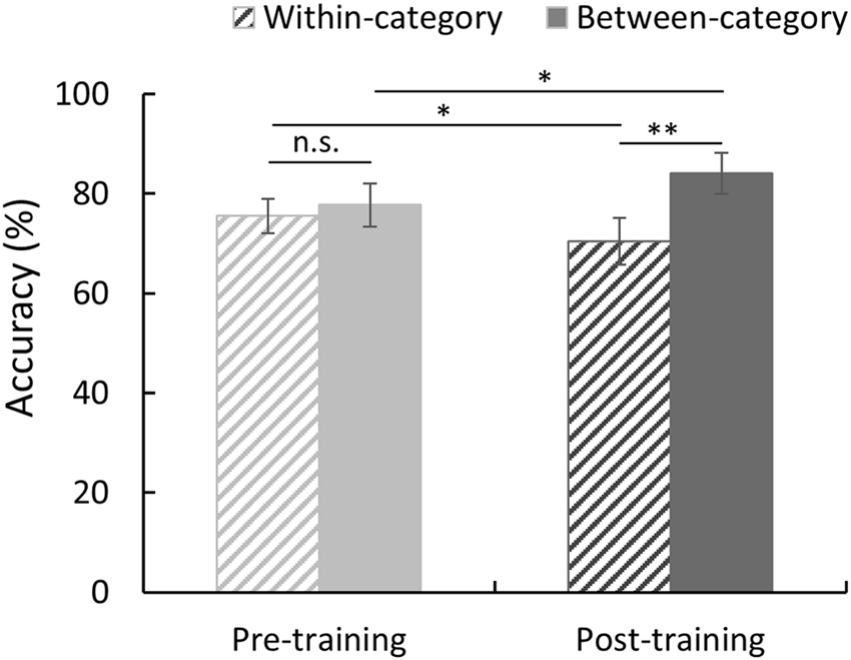
Participants’ mean accuracies for the within-category and between-category face pairs in the pre- and post-training AB-X discrimination tasks. Error bars represent the standard error of the mean. **p < 0.001; *p < 0.05; ns, not significant.

After the participants learned the new categories of the face stimuli, for Group 1, Face A and 50% B belonged to the same category, while Face B belonged to a different category; thus, the categorical boundary was between 50% B and Face B. The 5% B–30% B, 10% B–35% B, 15% B–40% B and 20% B–45% B pairs were the within-category pairs, and the 55% B–80% B, 60% B–85% B, 65% B–90% B and 70% B–95% B pairs were the between-category pairs. However, the categorical relationships between the face pairs acquired by Group 2 was opposite to that acquired by Group 1. Then, a two-factor repeated-measures ANOVA was performed and showed a significant interaction between the category relationship (between-category vs. within-category) and the training (pre- vs. post-training) (*F*(1, 22) = 8.419, *p* = 0.008, η_*p*_^2^ = 0.277; see Fig. 3). The post hoc paired t-tests showed that there was no significant difference between the discrimination accuracies of the within- and between-category stimuli in the pre-training test (*t*(22) = 0.984, *p* = 0.336; Cohen’s *d* = 0.21, 95% CI for the difference: −0.068 to 0.024), but the mean accuracy of the discrimination of the between-category pairs was significantly higher than that of the within-category pairs in the post-training test (*t*(22) = 4.334, *p* < 0.001, Cohen’s *d* = 0.90, 95% CI for the difference: 0.07 to 0.20). We also compared the mean accuracy in the pre- and post-training tests under each category condition. The participants’ discrimination of the morphed faces from the same learned category significantly decreased after the training (*t*(22) = 2.141, *p* = 0.044, Cohen’s *d* = 0.45, 95% CI for the difference: −0.10 to −0.002), while the discrimination of the between-category faces was significantly improved (*t*(22) = 2.083, *p* = 0.049, Cohen’s *d* = 0.43, 95% CI for the difference: 0.0003 to 0.127). The main effect of category was also significant (*F*(1, 22) = 18.090, *p* < 0.001, η_*p*_^2^ = 0.451), and there was no main effect of training (*F*(1, 22) = 0.103, *p* = 0.752, η_*p*_^2^ = 0.005). These results indicated that the learned categorical relationships modulated the individuals’ discrimination of faces, i.e., acquired distinctiveness for between-category faces and acquired equivalence for within-category faces.

### ERP data

Next, we measured the participants’ behavioral performance in circle fixation detection during the visual oddball task to examine their attention status. The proportion of correct hits was very high (M = 97.78%, SD = 4.65%), and the mean number of false alarm trials was less than two. The mean reaction time was 447.41 ms (SD = 41.65). These findings indicated effective attention in the face-irrelevant fixation detection task.

**Figure 4:**
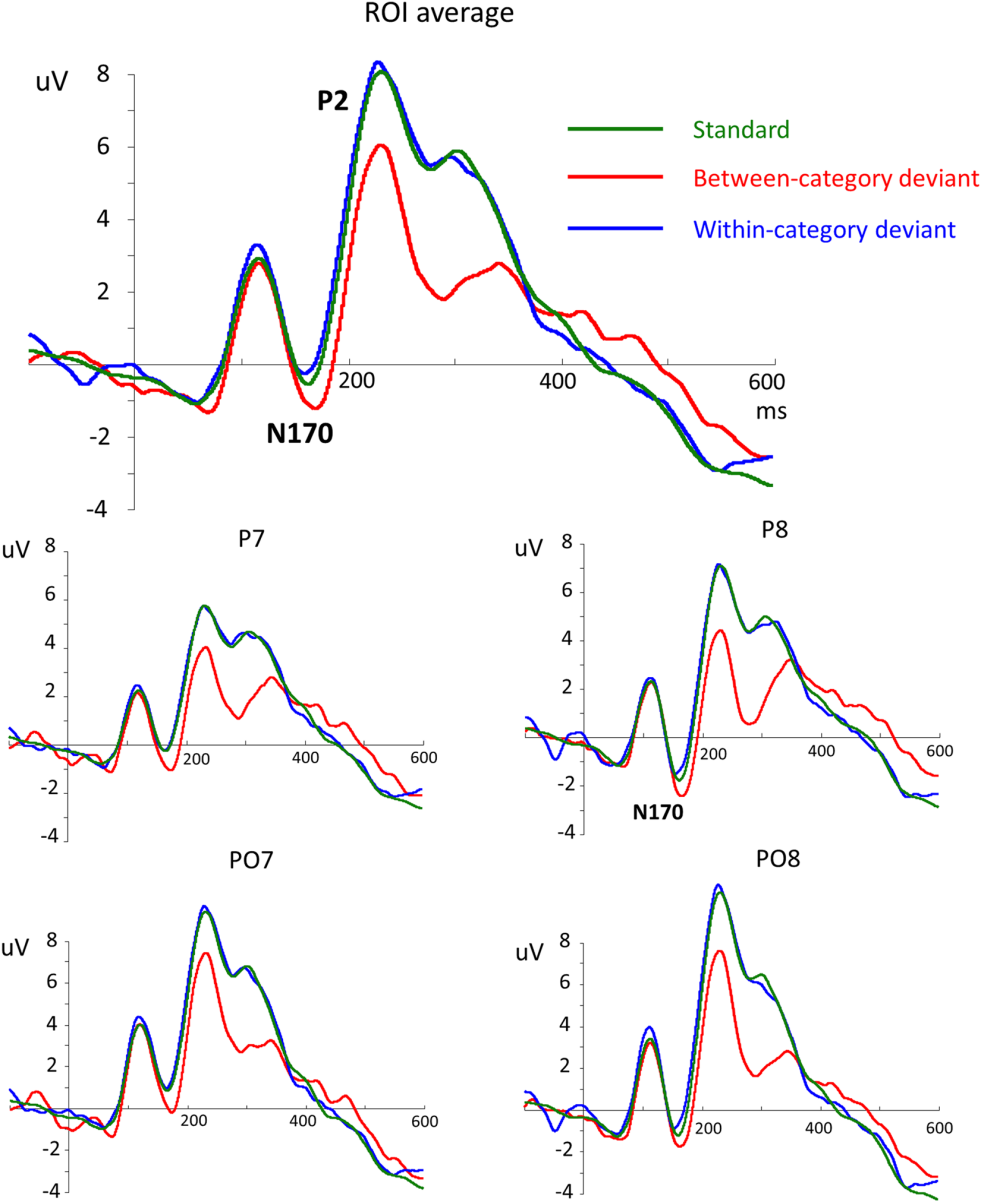
Grand average ERPs elicited by standard stimuli (green), between-category deviants (red) and within-category deviants (blue). Waveforms of ROI average depict a linear derivation of electrodes P5, P6, P7, P8, PO5, PO6, PO7 and PO8.

Then, we analyzed the ERP data that were collected during the post-training visual oddball task. Figure 4 illustrates the grand average waveforms that were elicited by the standard and deviant faces. At the posterior sites, the faces elicited N170 and subsequent P2 components. There was a significant main effect of the stimulus type on the N170 amplitudes (*F*(2,38) = 12.864, *p* = 0.001, η_*p*_^2^ = 0.404). The pairwise comparisons showed that the between-category deviant stimuli elicited larger N170s (−0.15 μV) than the within-category deviants (1.19 μV, *t*(19) = 3.741, *p* = 0.001, Cohen’s *d* = 0.84, 95% CI for the difference: −2.1 to −0.59) and the standard stimuli (0.824 μV, *t*(19) = 4.044, *p* = 0.001, Cohen’s *d* = 0.90, 95% CI for the difference: −1.48 to −0.47), and there was no significant difference between the within-category deviants and the standard stimuli (*t*(19) = 1.88, *p* > 0.05, Cohen’s *d* = 0.42, 95% CI for the difference: −0.04 to 0.77). Similarly, there was a significant main effect of stimulus type on the N170 peak latencies (*F*(2,38) = 6.194, *p* = 0.014, η_*p*_^2^ = 0.246). The pairwise comparisons showed that the between-category deviant stimuli elicited a delayed N170 (164.2 ms) compared to the within-category deviants (157.8 ms, *t*(19) = 2.66, *p* = 0.015, Cohen’s *d* = 0.59, 95% CI for the difference: 1.34 to 11.24) or standard stimuli (160.9 ms; *t*(19) = 2.48; *p* = 0.023, Cohen’s *d* = 0.55, 95% CI for the difference: 0.5 to 6.04), and there was no significant difference between the within-category deviants and the standard stimuli (*t*(19) = 2.01, *p* > 0.05, Cohen’s *d* = 0.45, 95% CI for the difference: −6.2 to 0.12). The larger delayed N170 that was elicited by the between-category deviants suggests that face CP was induced by the newly learned categories that occurred early in the time window of the N170.

Similar to the N170 component, there was a significant main effect of stimulus type on the P2 amplitudes (*F*(2,38) = 39.917, *p* < 0.001, η_*p*_^2^ = 0.678). The pairwise comparisons showed that the between-category deviants (3.81 μV) elicited a smaller P2 than the within-category deviants (6.68 μV, *t*(19) = 7.352, *p* < 0.001, Cohen’s *d* = 1.64, 95% CI for the difference: −3.66 to −2.05) and standard stimuli (6.47 μV, *t*(19) = 6.215, *p* < 0.001, Cohen’s *d* = 1.39, 95% CI for the difference: −3.56 to −1.76), and there was no significant difference between the within-category deviants and the standard stimuli (*t*(19) = 0.93, *p* > 0.05, Cohen’s *d* = 0.21, 95% CI for the difference: −0.26 to 0.67). There was a significant main effect of stimulus type on the P2 peak latencies (*F*(2,38) = 3.651, *p* = 0.047, η_*p*_^2^ = 0.161). The pairwise comparisons showed that the between-category deviant stimuli elicited an early P2 (235.4 ms) compared to the within-category deviant (245.3 ms, *t*(19) = 2.247, *p* = 0.037, Cohen’s *d* = 0.50, 95% CI for the difference: −19.13 to −0.68) or standard stimuli (241.4 ms, *t*(19) = 2.147, *p* = 0.045, Cohen’s *d* = 0.48, 95% CI for the difference: −11.82 to −0.15), but the difference between the within-category deviants and the standard stimuli was not significant (*t*(19) = 1.06, *p* > 0.05, Cohen’s *d* = 0.24, 95% CI for the difference: −3.83 to 11.66).

**Figure 5:**
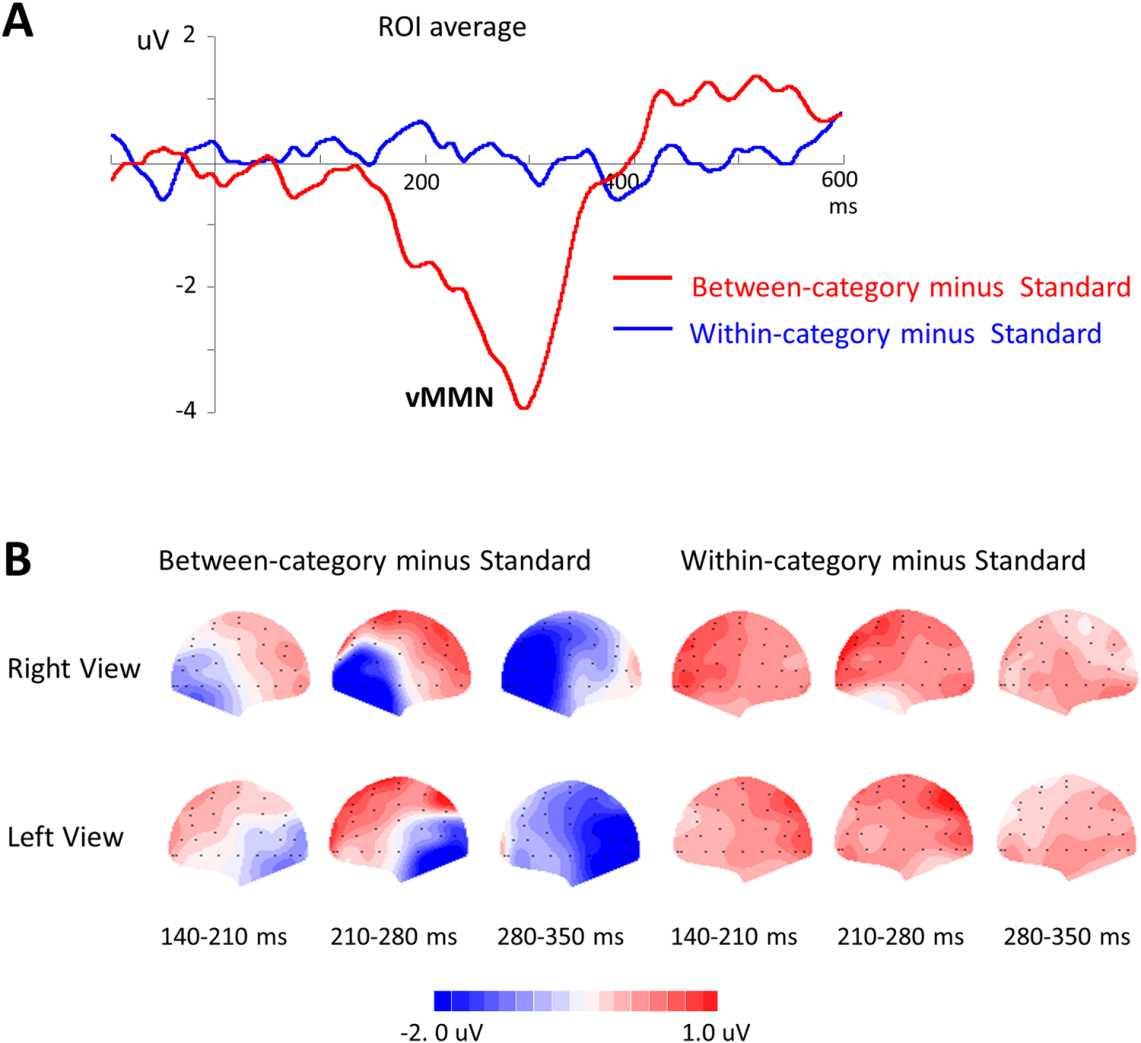
Grand-average vMMN. (A) The vMMN obtained by subtracting the ERPs for the standard stimuli from that for the between-category deviants (red) and that for the within-category deviants (blue). Waveforms depict a linear derivation of electrodes P5, P6, P7, P8, PO5, PO6, PO7 and PO8. (B) Scalp topographic distributions of the vMMN in consecutive 70 ms latency windows within 140- 350 ms latency range.

Next, we examined whether face CP could be observed by vMMN differences. The grand average difference waveforms, i.e., vMMNs, between the deviant and standard ERPs are depicted in Fig. 5. The vMMN that was elicited by the between-category deviant had a more evident negative deflection in the time range of 140–350 ms. One-sample t-tests and paired t-tests were performed to analyze the vMMN in the early (140–210 ms), middle (210–280 ms) and late latency ranges (280–350 ms). During each time range, the one-sample t-tests showed that the mean amplitudes of the between-category MMNs were significantly different from zero (*t*(19) = 4.44, *p* < 0.001, Cohen’s *d* = 0.99, 95% CI for the difference: −1.57 to −0.56; *t*(19) = 5.36, *p* < 0.001, Cohen’s *d* = 1.20, 95% CI for the difference: −3.45 to −1.51 and *t*(19) = 7.325, *p* < 0.001, Cohen’s *d* = 1.64, 95% CI for the difference: −3.61 to −2.00 in the early, middle, and late time ranges, respectively), whereas the difference wave between the ERPs in response to the within-category deviants and the standard stimuli did not differ from zero at any time range (all *p* > 0.05). The paired t-tests showed that the between-category deviant stimuli elicited a larger vMMN than the within-category deviant stimuli in each time range (*t*(19) = 4.427, *p* < 0.001, Cohen’s *d* = 0.99, 95% CI for the difference: −2.15 to −0.75; *t*(19) = 6.516, *p* < 0.001, Cohen’s *d* = 1.46, 95% CI for the difference: −3.57 to −1.83 and *t*(19) = 5.311, *p* < 0.001, Cohen’s *d* = 1.19, 95% CI for the difference: −3.85 to −1.67 in the early, middle, and late time ranges, respectively). These results indicate that face CP was induced by the newly learned categories and could occur pre-attentively during an early stage of processing.

## Discussion

To investigate whether newly learned categories could affect individuals’ perception of human faces, in the present study, we measured the participants’ behavioral performance in a face discrimination task and recorded the ERPs (i.e., N170 and vMMN) that were elicited by the face stimuli in the visual oddball task. After the participants learned the artificial face stimulus categories, that the behavior discrimination accuracy for face pairs from different acquired categories was significantly higher than that for pairs from the same category. Neurally, we found that the between-category deviant faces evoked larger and delayed N170s than the within-category deviants and standard stimuli. More importantly, the vMMNs obtained by the between-category deviants were stronger than those obtained by the within-category deviants. These results demonstrated that face perception could be modulated by newly learned categorical information, and this CP effect in faces occurred automatically during the pre-attentive processing stage.

In the discrimination task, the participants’ performance for the between-category faces was better than that for the within-category faces after the participants learned the artificial categories of the face stimuli. First, this result was consistent with previous findings that the CP effect was induced by newly learned categories in other domains. The perceptual discrimination of colors from different trained categories was significantly improved after participants learned to map the colors that were originally in the same lexical category (e.g., green) to different novel artificial categories via short-term intensive training^32^. Our study extended these findings by showing that CP was induced by newly learned categories and is not limited to low-level perceptual processing but can occur in higher-level face perception. Second, our finding that there was no CP effect in face discrimination before the training while CP occurred after the training was consistent with previous findings that no CP effect was observed for unfamiliar morphed faces without preexposure or with only a brief exposure to the endpoints of the continuum, but CP was induced when the endpoint faces were presented with clear category labels before the experiment^15^. Notably, after we controlled for the physical differences among the face stimuli and counterbalanced the category relationships of the stimuli across the participants, the observed discrimination difference between the face pairs after the training can only be explained by the effect of the learned categories. Thus, our study provides empirical evidence supporting that CP of faces was induced by short-term learned categories.

Our finding that between-category deviants elicited a larger N170 than the within-category deviants and standard faces was consistent with previous reports regarding the N170 in CP for facial expression^18^ and face identity^16,17^. This result was also consistent with previous findings that faces from other ethnic groups elicited more negative N170s, likely reflecting more effortful structural in encoding faces from different categories^35^. Additionally, consistently with the vMMNs elicited by changes in face orientation^28,29^ and facial expressions^31^, in the current study, the deviant faces whose acquired category different from that of the standard faces elicited evident vMMNs, starting from 140 ms after the stimuli onset. In the present study, compared with the responses to the standard stimuli, the face-specific N170 was significantly enhanced, and the P2 was significantly reduced at the occipital-temporal sites in response to the between-category deviants. Indeed, these changes in the N170 and P2 (i.e., more negative deflection in response to the between-category deviants) are not induced directly by face processing but rather reflect a vMMN induced by changes in faces from different categories. This interpretation is consistent with previous findings of face-vMMN in oddball tasks^28,36^. Importantly, our study extended previous findings by demonstrating the occurrence of CP of faces under the unattended condition. Moreover, the vMMN pattern in our study provided an ERP marker for face discrimination under the task-irrelevant condition.

In the present study, a larger vMMN was found in response to the deviant faces from different learned categories as standard faces. This result was consistent with previous electrophysiological findings that pre-attentive CP was induced by newly learned categories in the color and shape domains^27,33,37^, which may imply the generality of the CP effect as suggested by Gilbert *et al*.^38^. Furthermore, in the present study, a significant vMMN was observed by subtracting the ERP that was elicited by the standard stimuli from the between-category deviants; in contrast, this vMMN was not observed in response to the within-category deviants. According to Czigler *et al*., less salient deviants do not evoke a significant vMMN^21^, and our finding suggested that visual changes in the within-category faces were salient under the unattended condition than of the changes in the between-category faces after the participants acquired the different categorical information. Combined with the behavioral findings that discrimination performance for between-category faces was better during the post-training test than that during the pre-training test while the discrimination of the within-category faces was even worse, our results indicate that face CP could result from both acquiring distinctiveness for the between-category faces and acquiring equivalence for the within-category faces^39^, and CP may occur automatically during the early pre-attentive processing stage.

In summary, the present study demonstrated that face perception is modulated by newly learned categories, and this effect occurs unconsciously during an early stage of processing. There are several unaddressed issues that are important topics for future research. First, although our study provides evidence indicating that short-term learned categories affect face perception, it remains unknown when the CP effect occurs during the learning process. Future studies should test the CP effect after several minutes or every training session. Second, the N170 in our study was smaller than that of face CP in previous studies^16–18^, which may be a result of our paradigm, e.g., neural adaptation due to hundreds of repetitions. Studies using other tasks should further test the N170 effect in face CP that is induced by learned categories. Third, the results in the current study can’t test the specificity of CP in face perception. Since there is ample evidence that face processing is much different from other visual objects, it may be interesting to examine the face-specific CP in the future study. Finally, since the vMMN was associated with the automatic detection of changes in face perception in the present study, it could be used to test the interesting hypothesis that close relationships recreate the self^40^, e.g., by comparing the vMMNs elicited by faces of individuals in different relationships with the participant.

## References

[1] Bentin S, Allison T, Puce A, Perez E, McCarthy G (1996). Electrophysiological studies of face perception in humans. Journal of cognitive neuroscience.

[2] Liu J, Higuchi M, Marantz A, Kanwisher N (2000). The selectivity of the occipitotemporal M170 for faces. Neuroreport.

[3] Eimer M (2000). The face‐specific N170 component reflects late stages in the structural encoding of faces. Neuroreport.

[4] Bentin S, Deouell LY (2000). Structural encoding and identification in face processing: ERP evidence for separate mechanisms. Cognitive neuropsychology.

[5] Sagiv N, Bentin S (2001). Structural encoding of human and schematic faces: holistic and part-based processes. Journal of Cognitive Neuroscience.

[6] Zion-Golumbic E, Bentin S (2007). Dissociated neural mechanisms for face detection and configural encoding: evidence from N170 and induced gamma-band oscillation effects. Cerebral Cortex.

[7] Beale JM, Keil FC (1995). Categorical effects in the perception of faces. Cognition.

[8] Calder AJ, Young AW, Perrett DI, Etcoff NL, Rowland D (1996). Categorical perception of morphed facial expressions. Visual Cognition.

[9] Roberson D, Davidoff J (2000). The categorical perception of colors and facial expressions: The effect of verbal interference. Memory & Cognition.

[10] Kotsoni E, de Haan M, Johnson MH (2001). Categorical perception of facial expressions by 7-month-old infants. Perception.

[11] Fugate JM (2013). Categorical perception for emotional faces. Emotion Review.

[12] Levin DT, Angelone BL (2002). Categorical perception of race. Perception.

[13] Bülthoff I, Newell F (2004). Categorical perception of sex occurs in familiar but not unfamiliar faces. Visual Cognition.

[14] Levin DT, Beale JM (2000). Categorical perception occurs in newly learned faces, other-race faces, and inverted faces. Attention, Perception, & Psychophysics.

[15] Kikutani M, Roberson D, Hanley JR (2008). What’s in the name? Categorical perception for unfamiliar faces can occur through labeling. Psychonomic bulletin & review.

[16] Campanella S (2000). Right N170 modulation in a face discrimination task: an account for categorical perception of familiar faces. Psychophysiology.

[17] Jacques C, Rossion B (2006). The speed of individual face categorization. Psychological Science.

[18] Campanella S, Quinet P, Bruyer R, Crommelinck M, Guerit J-M (2002). Categorical perception of happiness and fear facial expressions: an ERP study. Journal of cognitive neuroscience.

[19] Czigler I (2007). Visual mismatch negativity: violation of nonattended environmental regularities. Journal of Psychophysiology.

[20] Czigler I, Balázs L, Pató LVG (2004). Visual change detection: event-related potentials are dependent on stimulus location in humans. Neuroscience letters.

[21] Czigler I, Balázs L, Winkler I (2002). Memory-based detection of task-irrelevant visual changes. Psychophysiology.

[22] Näätänen R, Jacobsen T, Winkler I (2005). Memory‐based or afferent processes in mismatch negativity (MMN): A review of the evidence. Psychophysiology.

[23] Kimura M, Katayama Ji, Ohira H, Schröger E (2009). Visual mismatch negativity: new evidence from the equiprobable paradigm. Psychophysiology.

[24] Thierry G, Athanasopoulos P, Wiggett A, Dering B, Kuipers J-R (2009). Unconscious effects of language-specific terminology on preattentive color perception. Proceedings of the National Academy of Sciences.

[25] Athanasopoulos P, Dering B, Wiggett A, Kuipers J-R, Thierry G (2010). Perceptual shift in bilingualism: Brain potentials reveal plasticity in pre-attentive colour perception. Cognition.

[26] Clifford A, Holmes A, Davies IR, Franklin A (2010). Color categories affect pre-attentive color perception. Biological psychology.

[27] Yu M, Mo C, Zeng T, Zhao S, Mo L (2017). Short-term trained lexical categories affect preattentive shape perception: Evidence from vMMN. Psychophysiology.

[28] Wang W, Miao D, Zhao L (2014). Automatic detection of orientation changes of faces versus non-face objects: a visual MMN study. Biological psychology.

[29] Wang W, Miao D, Zhao L (2014). Visual MMN elicited by orientation changes of faces. Journal of integrative neuroscience.

[30] Li X, Lu Y, Sun G, Gao L, Zhao L (2012). Visual mismatch negativity elicited by facial expressions: new evidence from the equiprobable paradigm. Behavioral and Brain Functions.

[31] Zhao L, Li J (2006). Visual mismatch negativity elicited by facial expressions under non-attentional condition. Neuroscience letters.

[32] Zhou K (2010). Newly trained lexical categories produce lateralized categorical perception of color. Proceedings of the National Academy of Sciences.

[33] Zhong W, Li Y, Li P, Xu G, Mo L (2015). Short‐term trained lexical categories produce preattentive categorical perception of color: Evidence from ERPs. Psychophysiology.

[34] Wang R, Li J, Fang H, Tian M, Liu J (2012). Individual differences in holistic processing predict face recognition ability. Psychological science.

[35] Wiese H, Kaufmann JM, Schweinberger SR (2014). The neural signature of the own-race bias: Evidence from event-related potentials. Cerebral Cortex.

[36] Stefanics G, Csukly G, Komlósi S, Czobor P, Czigler I (2012). Processing of unattended facial emotions: a visual mismatch negativity study. Neuroimage.

[37] Mo L, Xu G, Kay P, Tan L-H (2011). Electrophysiological evidence for the left-lateralized effect of language on preattentive categorical perception of color. Proceedings of the National Academy of Sciences.

[38] Gilbert AL, Regier T, Kay P, Ivry RB (2008). Support for lateralization of the Whorf effect beyond the realm of color discrimination. Brain and language.

[39] Goldstone RL (1994). Influences of categorization on perceptual discrimination. Journal of Experimental Psychology: General.

[40] Aron A (2004). Including others in the self. European review of social psychology.

